# Different Effects of Riociguat and Vericiguat on Pulmonary Vessels and Airways

**DOI:** 10.3390/biomedicines13040856

**Published:** 2025-04-02

**Authors:** Katharina Nubbemeyer, Julia Krabbe, Svenja Böll, Anna Michely, Sebastian Kalverkamp, Jan Spillner, Christian Martin

**Affiliations:** 1 Department of Thoracic Surgery, Medical Faculty, RWTH Aachen University, Pauwelsstraße 30, 52074 Aachen, Germany; knubbemeyer@ukaachen.de (K.N.); skalverkamp@ukaachen.de (S.K.); jspillner@ukaachen.de (J.S.); 2 Institute for Prevention and Occupational Health Bochum (IPA), Ruhr University Bochum, Bürkle de la Camp-Platz 1, 44789 Bonn, Germany; julia.krabbe@dguv.de; 3 Department of Pediatrics, Medical Faculty, RWTH Aachen University, Pauwelsstraße 30, 52074 Aachen, Germany; svenja.boell@gmx.de (S.B.); amichely@ukaachen.de (A.M.); 4 Institute of Pharmacology and Toxicology, Medical Faculty, RWTH Aachen University, Wendlingweg 2, 52074 Aachen, Germany

**Keywords:** precision cut lung slices, PCLS, rat, riociguat, vericiguat, pulmonary hypertension, soluble guanylate cyclase

## Abstract

**Background/Objectives**: Pulmonary hypertension is a progressive disease leading to right heart failure. One treatment strategy is to induce vasodilation via the nitric oxide–soluble guanylate cyclase–cyclic guanosine monophosphate (NO–sGC–cGMP) signaling pathway. There are currently two soluble guanylate cyclase stimulators on the market: Riociguat and vericiguat, with vericiguat having a longer half-life and needing to be taken only once a day. This study investigated whether the pharmacological differences between the drugs affect pulmonary vessels and airways. **Methods**: The effects of vericiguat and riociguat on pulmonary arteries, veins, and airways were studied using rat precision-cut lung slices (PCLS). Vessels were pretreated with endothelin-1 and airways with serotonin. In isolated perfused lungs (IPL), the effects of sGC stimulation on pulmonary artery pressure (PAP), airway resistance, inflammatory cytokine, and chemokine release were quantified. **Results**: Riociguat and vericiguat caused pulmonary artery dilation in PCLS. During IPL, riociguat was more effective than vericiguat in reducing PAP with a statistically significant reduction of 10%. Both drugs were potent bronchodilators in preconstricted airways (*p* < 0.001). Only vericiguat reduced airway resistance during IPL, as shown here for the first time. Both drugs significantly reduced IL-6 and IL-1ß levels, while riociguat also reduced VEGF-A and KC-GRO levels. **Conclusions**: Riociguat and vericiguat had three main effects in the two rat ex-vivo models: They dilated the pulmonary arteries, induced bronchodilation, and reduced inflammation. These properties could make sGC stimulators useful for treating diseases associated with endothelial dysfunction. In the future, vericiguat may provide an alternative treatment to induce bronchodilation in respiratory diseases.

## 1. Introduction

Pulmonary hypertension (PH) is a progressive disease that leads to right heart failure and has a poor prognosis if left untreated [1]. The pathogenesis of pulmonary hypertension is multifactorial, with remodeling of peripheral pulmonary arteries and perivascular inflammation, causing endothelial dysfunction, proliferation of smooth muscle cells, and fibroblasts [2]. In patients with chronic thromboembolic pulmonary hypertension (CTEPH), a subtype of PH, inflammatory thrombosis, and deficient angiogenesis contribute to its development [3]. Recent research reports elevated pro-inflammatory cytokines TNFα, IL-1ß, IL-6, and IL-8 in PH patients [4,5,6]. The levels of IL-6 correlate with the disease severity, prognosis, and treatment effect in patients with CTEPH [7]. The angiogenesis in patients with CTEPH is driven by the vascular endothelial growth factor (VEGF), functioning via the nitric oxide–soluble guanylate cyclase–cyclic guanosine monophosphate (NO-sGC-cGMP) pathway [8,9], and leading to the dissolution of thrombi and neovascularization [3].

One primary treatment strategy of PH is the induction of vasodilation via the NO-cGMP-sGC signaling pathway. In brief, nitric oxide (NO) binds to the reduced heme group (Fe^2+^) of soluble guanylate cyclase (sGC), causing the production of cyclic guanosine monophosphate (cGMP) in vascular smooth muscle cells. As a second messenger, cGMP activates protein kinase G and dephosphorylates actin-myosin, resulting in vascular relaxation [10]. For this purpose, drugs have been developed to directly stimulate sGC. Their mechanism of action depends on a reduced heme group and can occur in two ways. They can enhance the binding of NO and act synergistically. Additionally, they can directly stimulate sGC in the absence of NO [11]. In 2013, riociguat (BAY 63-2521) became the first soluble guanylate cyclase stimulator to be clinically approved for the treatment of primary and chronic thromboembolic pulmonary hypertension [12,13]. The randomized, double-blind, phase III clinical trial PATENT-1 demonstrated that riociguat improved 6 min walk distance and hemodynamic parameters given at a dose of up to 2.5 mg three times daily [13]. Vericiguat (BAY 102-1189) is a next-generation compound with a longer half-life, requiring only a single daily dose [14]. It is considered a suitable treatment for cardiovascular disease [14], as it has been shown to reduce the risk of death and the need for hospitalization in patients with heart failure with reduced ejection fraction [15]. At present, only riociguat and vericiguat have been approved for clinical use as sGC stimulators.

Morbidelli et al. showed that sGC inhibitors reduce angiogenesis, suggesting that sGC stimulators promote pro-angiogenic effects [8]. To date, only a limited number of anti-inflammatory and pro-angiogenic effects of sGC stimulators have been identified in the existing scientific literature. sGC stimulators act anti-inflammatory by inhibiting leukocyte rolling and adhesion [16], a reduction in eosinophils has been described in asthma [17].

Furthermore, the effects of riociguat and vericiguat on the pulmonary veins are not yet fully elucidated, despite their clinical relevance. The pulmonary veins contribute to pulmonary vascular resistance and left heart preload [18]. According to Dardi et al., NO is a significant endothelium-dependent relaxing factor in veins [19]. However, the effects of sGC stimulators on pulmonary veins have not been investigated.

The NO-sGC-cGMP pathway can mediate not only vasodilation but also bronchodilation. Bronchodilation is an important therapeutic strategy in respiratory diseases like asthma. To date, there have been a few experimental studies that suggest a bronchodilating effect of sGC stimulators [20,21].

This study hypothesized distinct effects of riociguat and vericiguat on pulmonary arteries, veins, and bronchi due to their pharmacological differences, tested by precision-cut lung slices (PCLS) and isolated lung perfusion (IPL). The following effects were investigated: 1. The efficacy of riociguat and vericiguat in inducing vasodilation of pulmonary arteries and veins, 2. The efficacy in inducing bronchodilation, and 3. Their anti-inflammatory properties.

## 2. Materials and Methods

Precision-cut lung slices: Wistar rats (283 ± 39 g) of both sexes were obtained from Janvier Labs (Le Genest-Saint-Isle, France) and used as donor lungs. Experiments were performed in accordance with the European Parliament Directive 2010/63/EU and approved by the Animal Welfare Department of the Institute of Laboratory Animal Science of the University Hospital of RWTH Aachen University (ID: 40143A4).

Rat PCLS were obtained from Wistar rats as previously described [22,23]. Intraperitoneal anesthesia was induced by administration of 600 mg/kg sodium pentobarbital (Narcoren, Merial GmbH, Hallbergmoos, Germany). After the death of the animal, a tracheotomy was performed, followed by a sternotomy and cannulation of the pulmonary artery. The pulmonary vessels were filled with warm gelatin, and the lungs with warm 1.5% low-melting agarose (both from Sigma-Aldrich Chemie GmbH, Steinheim, Germany. The lungs were cooled with ice to solidify the agarose. Once the agarose had hardened, the lung was removed. The lobes were separated, and 10 mm tissue cores were punched out using a sharpened metal tube. Visual inspection was performed to ensure that the cores contained centred airways or vessels. The experiments were conducted on large bronchi up to the 8th generation and on lobar, segmental, and subsegmental pulmonary arteries. Using a microtome (Alabama R&D Tissue Slicer MD6000, Alabama Research and Development, Munford, AL, USA), the cores for lungs were sliced to a thickness of 250–300 µm. The slices were placed in Petri dishes containing warmed culture medium and incubated at 37 °C in a humidified atmosphere containing 5% carbon dioxide. The culture and slicing medium have been prepared as previously described [24,25]. Measurements were performed on the following 2 days. However, this timeframe may not capture long-term drug effects. The medium was changed every day.

**Figure 1:**
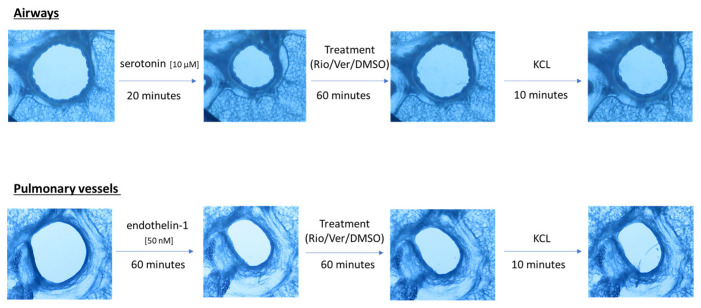
Schematic of the PCLS rat experiment procedure.

Airway and vessel diameters were recorded using a microscope (Scientific Workshops, RWTH Aachen University, Aachen, Germany) with a commercially available USB camera attached. One PCLS was used per vessel or airway. The change in diameter was analyzed using ImageJ 1.4.3 (U.S. National Institutes of Health, Bethesda, MD, USA). The area of the airway or vessel was defined as 100% before the addition of any drug. To test the effect of riociguat (BAY 63-2521) (Cayman Chemicals, Ann Arbor, MI, USA) and vericiguat (BAY 102 1189) (AChemBlock, Hayward, CA, USA) on airways and vessels, preconstriction was performed. Airways were preconstricted with serotonin (10 µM) in rat PCLS (from Sigma-Aldrich Chemie GmbH, Steinheim, Germany). Endothelin-1 (50 nM) (Bachem AG, Bubendorf, Switzerland) was added to induce vasoconstriction. Images were taken at baseline and after the addition of the pretreatment. Thereafter, images were acquired every 5 s for the airways and every 30 s for the vessels. After incubation with the pretreatment for 20 min in the airways and 60 min in the vessels, the treatment was added to induce dilation. There were two treatment groups with riociguat or vericiguat at three different concentrations (1 µM, 10 µM, 32 µM), and a control group with the vehicle dimethyl sulfoxide (DMSO). Again, images were taken after treatment. Dilation or constriction of bronchi and vessels was expressed as a percentage of the initial airway area (IAA) or initial vessel area (IVA). At the end of the experiment, KCL was used to demonstrate the vitality of the lung slices (Figure 1). The bronchi could be identified microscopically by their ciliated epithelium. Pulmonary arteries could be distinguished from veins due to their proximity to the bronchi.

Isolated lung perfusion: Isolated lung perfusion (IPL) was performed as previously described [26]. Briefly, the rats received 600 mg/kg sodium pentobarbital. After death and tracheal cannulation, positive pressure ventilation was started (70 breaths/min). A sternotomy and cannulation of the pulmonary artery and left atrium were performed. Perfusion was started with a constant flow of a recirculating perfusion solution. The pH was maintained between 7.1 and 7.4 by gassing with CO_2_. After the establishment of a perfusion circuit, the lungs and heart were transferred to the negative pressure chamber. Ventilation was performed between −12 and −4 cm H_2_O. A deep breath was taken every 5 min to prevent atelectasis. The perfusion flow was increased to a constant flow of 24 mL/min. Weight measurements were made continuously with an integrated dynamometer. All data were recorded and analyzed using Pulmodyn Software 2.0 (Hugo Sachs Elektronik, March-Hugstetten, Germany). After 30 min, endothelin-1 (5 nM) was added to increase the pulmonary artery pressure. A plateau of effect was seen after further 60 min of perfusion. At this point, one of the two treatments (riociguat/vericiguat) or the control was added at a concentration of 1 µM. After 30 min, the concentration was increased to 10 µM and after a further 30 min to 32 µM. Perfusion was performed for a total of 180 min. After 180 min, the IPL was stopped, and the right upper lobe was removed and weighed. To determine the W/D ratio, the tissue was dried at 65 °C for 48 h, and the dry weight was measured.

Enzyme-linked immunosorbent assays: Perfusate samples were taken at three different times during isolated lung perfusion: At the beginning of the perfusion, after the addition of endothelin, and at the end of the experiment after the addition of the treatment. The levels of cytokines IL-1ß, IFN-y, IL-4, IL-5, IL-6, IL-10, IL-13, keratinocyte chemoattractant/human growth-regulated oncogene (KC/GRO) and tumor necrosis factor alpha (TNF-α) were measured in perfusate samples using the MSD^®^ (Meso Scale Discovery^®^, Rockville, MD, USA) V-Plex Pro-Inflammatory Panel 2 (rat) kits according to the manufacturer′s instructions. The levels of monocyte chemoattractant protein 1 (MCP-1) and vascular endothelial growth factor (VEGF-A) were determined using the MSD^®^ U-Plex multiplex assay kit.

Statistical analysis: Quantitative results are presented as means ± standard error of the mean (SEM). Statistical analysis was performed using a general linear mixed model (GLMM) analysis (PROC GLIMMIX, SAS 9.4, SAS Institute Inc., Cary, NC, USA) assuming a normal distribution (lung function parameters of ILP, cytokines/chemokines), and beta distribution (change of initial area in precision-cut lung slices) of the data. GLMM analysis included treatment groups, time points, and inter-animal variability. In case of heteroscedasticity (based on covtest statement), the degrees of freedom were adapted by the Kenward-Roger approximation. A covariate analysis was performed to consider the effect of endothelin-1 in isolated perfused lungs and, in addition, the effect of KCL in PCLS on the results as covariates. If the covariate had an effect on the groups, an ANCOVA was performed. Multiple comparisons were corrected using the Shaffer-simulated (SIM) stepdown procedure. *p*-values < 0.05 were considered significant (* *p* < 0.05, ** *p* < 0.01, *** *p* < 0.001). Wet/Dry Ratio data were analyzed for the optimal distribution using the Shapiro–Wilk test (GraphPad Prism 9). If data did not show normal distribution, a non-parametric Kruskal–Wallis test was performed (GraphPad Prism 9). Multiple comparisons were corrected by false discovery rate. Outlier analysis was performed using an outlier calculator based on the Grubbs test (GraphPad) with an alpha level of significance of 0.05.

## 3. Results

### 3.1. Precision Cut Lug Slices

#### 3.1.1. Pulmonary Arteries and Veins

The effects of stimulators of soluble guanylate cyclase were investigated in endothelin-pretreated PCLS. Following the addition of endothelin-1, the pulmonary arteries were constricted to 55.19 ± 2.36% of initial vessel area (IVA) and the pulmonary veins to 48.71% ± 3.56% of IVA.

**Figure 2:**
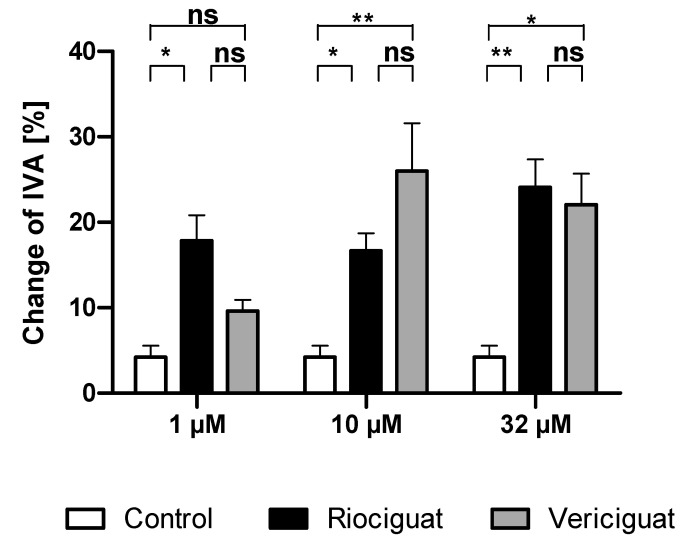
Riociguat and vericiguat cause vasodilation in preconstricted pulmonary arteries. Preconstriction was performed with endothelin-1 (50 nM) for 60 min in rat PCLS, followed by the addition of treatment for another 60 min. (1 μM): *n* = 7 riociguat, *n* = 8 vericiguat, and *n* = 8 control; (10 μM): *n* = 8 in all groups; (32 μM): *n* = 9 riociguat, *n* = 10 vericiguat, *n* = 8 control. * *p* < 0.05, ** *p* < 0.01, ns = not significant. Abbreviation is IVA = Initial vessel area.

Only riociguat (Rio) had a significant dilatory effect on pulmonary arteries at the lowest concentration (1 µM) in comparison to the control group. Application of vericiguat (Ver) did not result in significant vasodilation. At higher concentrations (10 µM and 32 µM), both riociguat and vericiguat caused significant dilation compared to the control. However, there was no statistically significant difference between the two drugs in all concentrations (Figure 2, Table 1).

**Figure A1:**
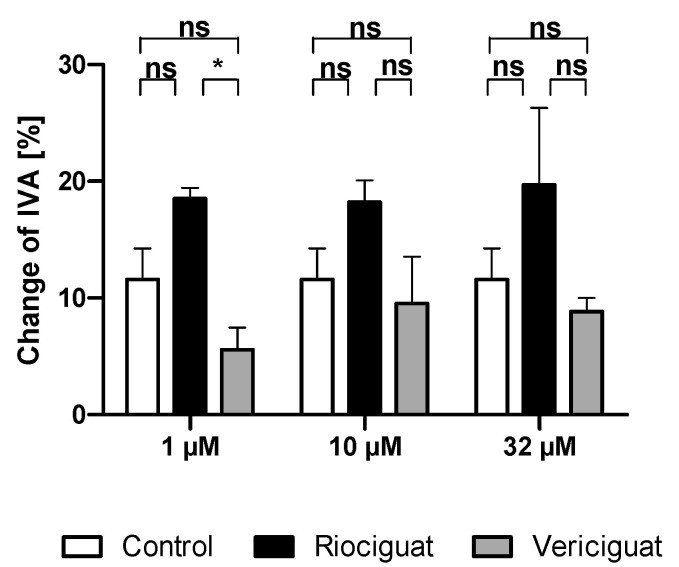
No significant vasodilatation was seen in pulmonary veins compared to the control group. Rat PCLS were preconstricted with endothelin-1 (50 nM), and treatments were added. (1 μM): n = 5 riociguat; *n* = 6 vericiguat; *n* = 4 control; (10 μM): *n* = 4 riociguat; *n* = 5 vericiguat; *n* = 4 Control; (32 μM): *n* = 5 riociguat; *n* = 4 vericiguat; *n* = 4 control. *p* < 0.05 is considered as significant: (* *p* < 0.05, ns = not significant). Abbreviation is IVA = Initial vessel area.

Both riociguat and vericiguat were observed to cause slight venous dilation. This effect was not statistically significant at any concentration when compared to the control group (all *p* > 0.05, Appendix A.1, Figure A1). At the lowest concentration of 1 µM, the administration of riociguat resulted in a significant venodilation compared to vericiguat (*p* = 0.028), however, this effect was not statistically significant compared to the control group (*p* > 0.05).

#### 3.1.2. Airways

**Figure 3:**
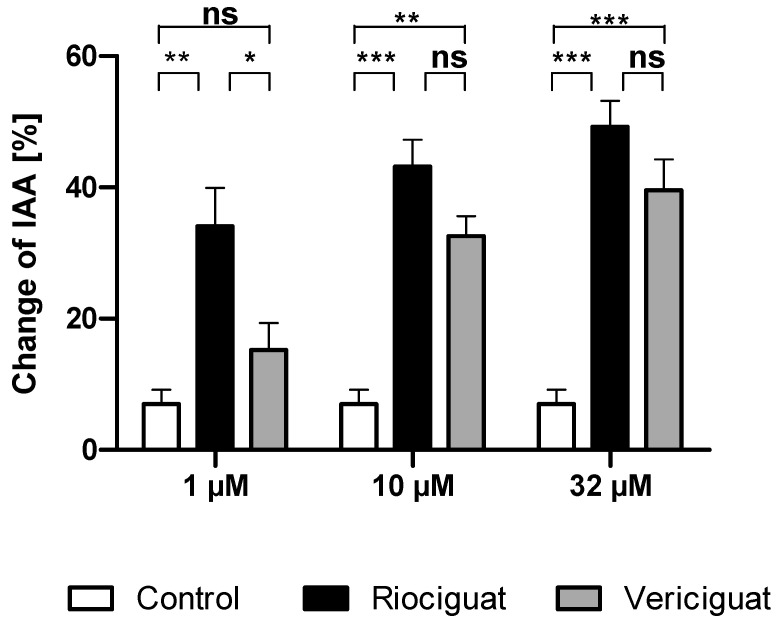
Riociguat and vericiguat cause bronchodilation in airways. PCLS of the rat were preconstricted with serotonin (10 μM), followed by treatment with sGC stimulators riociguat and vericiguat and the vehicle DMSO as a control group. Treatment concentrations (1 μM and 10 µM): *n* = 11 control, *n*= 9 riociguat, *n* = 8 vericiguat; (32 µM): *n* = 11 control, *n* = 11 riociguat, *n* = 8 vericiguat. The graph represents means ± SEM, * *p* < 0.05, ** *p* < 0.01, *** *p* < 0.001, ns = not significant. Abbreviation is IAA = Initial airway area.

Next, we studied whether the dilatory effect of soluble guanylate cyclase stimulators is also observable in the airways. To this end, the airways were contracted to 32.90 ± 2.15% of initial airway area (IAA) by serotonin. The degree of precontraction was included as a covariate in the statistical analysis and affected the different treatment groups. At the lowest concentration of 1 µM, riociguat dilated the airways significantly compared to the control group and to vericiguat. In contrast, vericiguat did not cause any significant dilation effect. At higher concentrations of 10 µM and 32 µM, both treatment groups exhibited a bronchodilatory effect compared to the control. There was no significant difference between the two treatment groups (Table 2 and Figure 3).

### 3.2. Isolated Lung Perfusion

#### 3.2.1. Pulmonary Artery Pressure

**Figure 4:**
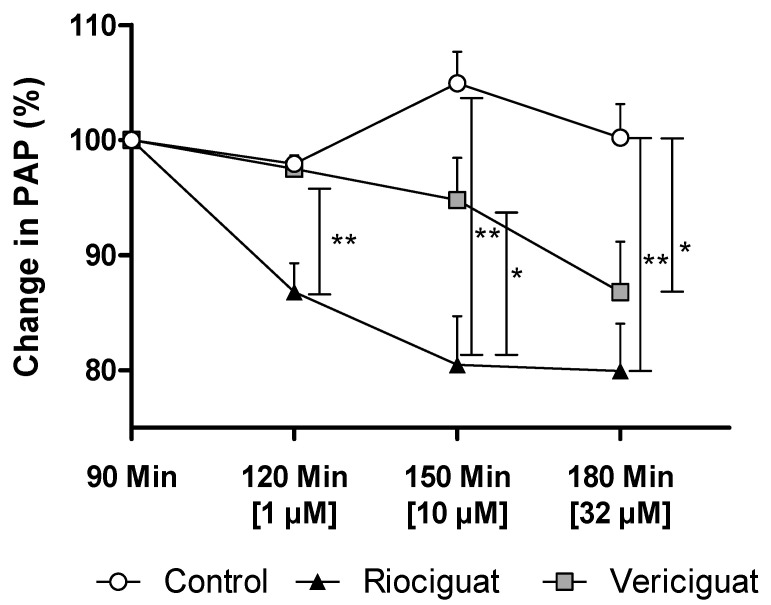
Vericiguat and riociguat cause significant vasodilation during isolated lung perfusion. The graph shows the percentage change in PAP after administration of endothelin-1 and treatment with different guanylate cyclase stimulators. Treatment groups: Control (*n* = 6); riociguat (*n* = 8), vericiguat (*n* = 7). Data are expressed as mean ± standard error of the mean (SEM). Statistical analysis was performed by using the absolute values. *p* < 0.05 is considered as significant: * *p* < 0.05, ** *p* < 0.01. Abbreviation is PAP = pulmonary artery pressure.

In precision-cut lung slices, the vasodilatory effect of both guanylate cyclase stimulators on pulmonary arteries was demonstrated. In order to ascertain whether these drugs retain their efficacy in an intact organ under perfusion and ventilation, IPL was used for a more physiologically relevant assessment than static PCLS. The pretreatment with endothelin-1 (final concentration in the buffer: 5 nM) increased PAP by 5.63 ± 0.55 cm H_2_O (baseline 4.67 ± 0.59 cm H_2_O), reaching a plateau after 60 min. At concentrations of 1 µM and 10 µM, only riociguat demonstrated a significant dilatory effect, resulting in a decrease in PAP compared to vericiguat and the control group (Table 3). When expressed as a percentage, the results of the study demonstrate a 10.7% reduction in pulmonary artery pressure treated with riociguat (1 µM) compared to vericiguat (1 µM) and the control (Figure 4). At higher concentrations, a significant difference was observed, with a >14% greater reduction in PAP with riociguat (10 µM), compared to vericiguat (10 µM) and the control. At the highest concentration (32 µM), both riociguat and vericiguat demonstrated a significant dilatory effect with no significant difference between the two drugs.

#### 3.2.2. Airway Resistance

**Figure 5:**
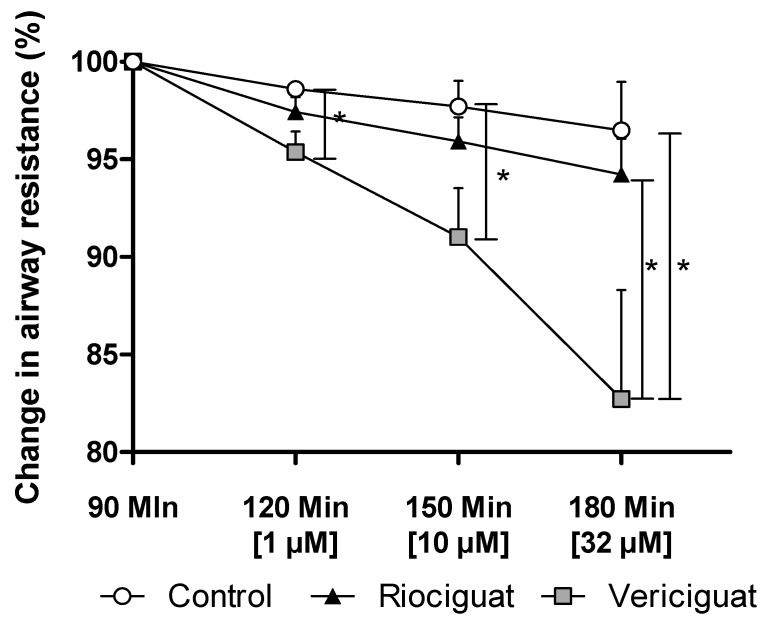
Vericiguat reduces airway resistance during isolated lung perfusion. The graph illustrates the percentage change in airway resistance following the administration of endothelin-1 and treatment with the guanylate cyclase stimulators. Treatment groups: Control (*n* = 5); Riociguat (*n* = 8); Vericiguat (*n* = 7). Data are expressed as mean ± standard error of the mean (SEM). *p* < 0.05 is considered as significant: (* *p* < 0.05).

Only vericiguat showed a significant decrease in airway resistance at all concentrations (Figure 5). At the highest concentration used (32 µM), there was a significant difference between vericiguat and riociguat (Table 4).

#### 3.2.3. Wet/Dry Ratio

**Figure A2:**
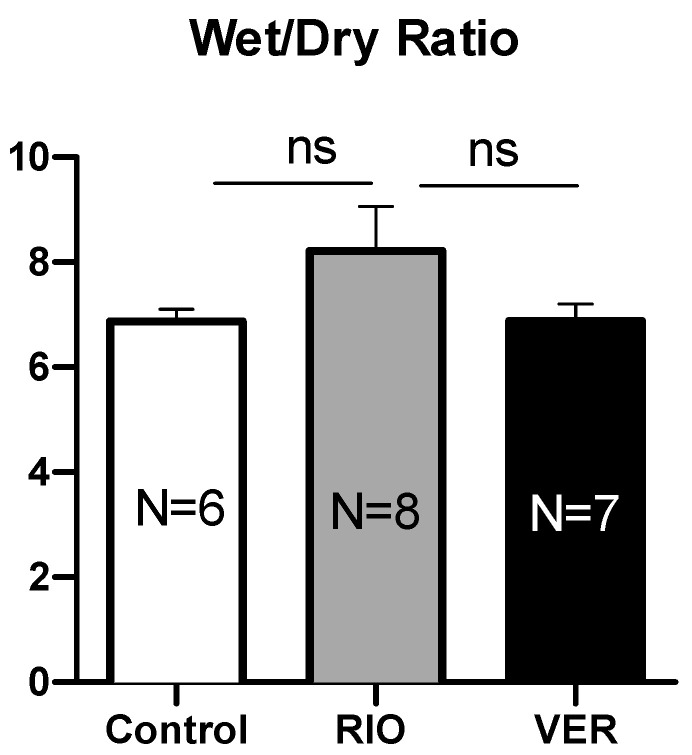
The treatments did not result in a discernible impact on the wet/dry ratio. Wet/Dry ratio: Riociguat 8.22 ± 0.84 vs. vericiguat 6.88 ± 0.32 vs. control group 6.87 ± 0.23. *p <* 0.05 is considered significant, ns = not significant. Abbreviations are RIO = Riociguat, VER = Vericiguat.

To investigate the effect of the drugs on the development of pulmonary oedema, the Wet/Dry ratio was measured. There were no significant differences between the groups (Appendix A.2, Figure A2).

#### 3.2.4. Cytokines and Chemokines

**Figure 6:**
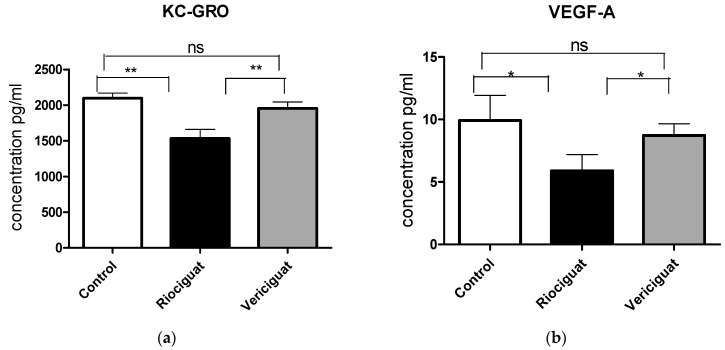

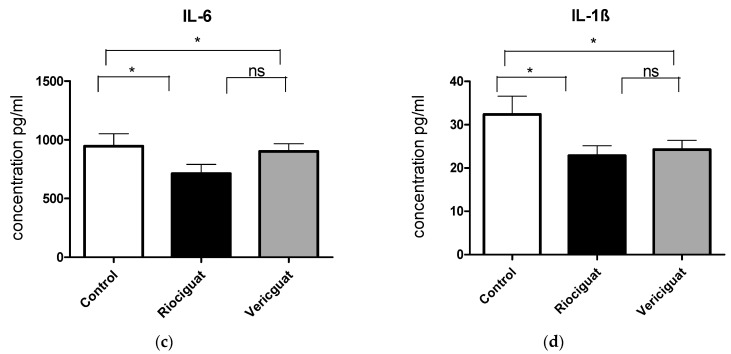
The bar charts illustrate the impact of sGC stimulators on (**a**) the levels of KC-GRO, (**b**) the levels of VEGF-A, (**c**) the levels of IL-6, and (**d**) the levels of IL-1ß. Treatment groups: Control group (*n* = 6); Riociguat (*n* = 8); Vericiguat (*n* = 7). Data are expressed as mean ± standard error of the mean (SEM). *p* < 0.05 is considered as significant: * *p* < 0.05, ** *p* < 0.01, ns = not significant.

As cytokines and chemokines play a crucial role in the pathogenesis of PH, the next step was to examine the effect of the drugs by analyzing the perfusate. Riociguat significantly reduced the levels of KC-GRO and VEGF-A in the perfusate compared to the control and to vericiguat (Figure 6a,b). Vericiguat had no effect on KC-GRO and VEGF-A levels. Both drugs significantly reduced IL-6 and IL-1ß levels compared to the control group (Figure 6c,d). There was no significant difference between the two treatment groups (Table 5). Application of vericiguat or riociguat had no significant effect on cytokine levels of IL-4, IL-5, IL-10, IL-13, TNFα, MCP-1, or IFNγ (Table S1).

## 4. Discussion

The present study investigated the impact of pharmacological differences between riociguat and vericiguat on the treatment of PH. It demonstrated that both drugs induce pulmonary arterial vasodilation, with riociguat being more effective, as shown here for the first time in a direct comparison using the same animal model. The results obtained for riociguat confirm its bronchodilator and anti-inflammatory effects by reducing IL-6 and IL-1ß levels. However, the underlying molecular mechanisms remain unclear. In addition, a novel effect of riociguat was observed: the reduction of KC-GRO and VEGF-A levels. The reduction in VEGF-A seems to contradict previously reported results. Moreover, this study provides novel pharmacological properties of vericiguat, including anti-inflammatory effects and reduced airway resistance during isolated lung perfusion, suggesting new potential applications for the drug.

In a first step, the effects of riociguat and vericiguat on pulmonary arteries in PLCS were investigated. So far, riociguat and vericiguat have been analyzed separately in different models [27,28], making it difficult to draw conclusions about their differences. This study was the first to directly compare the two sGC stimulators using the same animal model. Both drugs induced significant pulmonary artery dilation with no statistical difference in PCLS. PCLS allows studies on living tissue over several days with reliable results [29]. However, as PCLS is an experimental model without circulation, IPL was performed as a proof of principle. IPL has the advantage of applying precise drug concentrations under controlled ventilation and perfusion. During IPL, riociguat reduced PAP by more than 10% compared to vericiguat. Riociguat is therefore more effective than vericiguat in reducing PAP as presented here. Vericiguat has been developed to overcome the major disadvantage of riociguat, its short half-life [14]. It has an additional fluorine group, resulting in a prolonged half-life of approximately 30 h [14,30]. In 2021, it was approved for the treatment of cardiovascular disease [30]. There are few studies investing in the effects of vericiguat on pulmonary artery dilation [27,31]. The results presented here are in alignment with these findings, as only a slight decrease in PAP after administration of vericiguat was measured. The authors suggested that riociguat and vericiguat have different affinities for vascular and cardiac soluble guanylate cyclase [27]. The perfusion flow during IPL, but also during in vivo studies, generates shear stress and thus induces the release of NO in the endothelium [32]. Since sGC stimulators are known to enhance NO binding [33], the results may indicate that riociguat exerts a stronger synergistic effect in the presence of NO than vericiguat. This hypothesis is supported by the finding that in in vitro PCLS experiments, both drugs induced pulmonary arterial vasodilation, but in the IPL setting, riociguat was more potent. This effect may also be clinically relevant for patients, as the wall tension of the pulmonary arterioles [34] is the primary cause of pulmonary vascular resistance (PVR). Consequently, the administration of riociguat may lead to a substantial reduction in afterload of the right heart.

Although the pulmonary veins contribute to PVR, the effect of sGC stimulators on them is unknown. Previous research has suggested that sGC stimulators cause vasodilation due to high concentrations of eNOS and sGC in the vein wall [35]. However, the current study showed for the first time that neither drug had a significant effect on the pulmonary veins in PCLS. Riociguat and vericiguat led to mild venous dilation, though this was not statistically significant compared to the control group. This may be due to transient endothelin-1 effects or venous reactivity differences. Nevertheless, the veins exhibited a comparable response to sGC stimulators as the arteries did. Further experiments in animal models of PH induced by chronic hypoxia or monocrotaline are of great interest.

In addition, we investigated the effect of riociguat and vericiguat on the airways, as there are only a few studies on this topic [20,21,36,37]. The evidence presented here demonstrates that riociguat and vericiguat are potent bronchodilators in preconstricted healthy airways in PCLS (*p* < 0.001). The bronchodilatory effect of riociguat is consistent with previous studies [20,21]. During isolated lung perfusion, riociguat had no impact on airway resistance. Contrary to this outcome, the intratracheal administration of BAY 41-2272 (an analogue of riociguat) led to a reduction in airway resistance in asthmatic mice [20], whereas BAY 41-2272 showed no effect on the airway resistance during whole-body plethysmography in a model of COPD [37]. In airways, NO is produced continuously, consequently, exogenous NO does not induce additional bronchodilation [20]. Direct stimulation of sGC increases cGMP production and could provide bronchodilatory effects even in the presence of NO [20]. In the present study, healthy lungs with endothelin-1-induced bronchoconstriction were analyzed. It was hypothesized that baseline levels of NO and cGMP may differ between healthy and diseased lungs, possibly affecting the effect of riociguat in different experimental settings. This study on intact organs demonstrated a statistically significant reduction in airway resistance in the vericiguat group. To our knowledge, this is the first study on the effect of vericiguat on airway resistance that provides new insights into the pharmacological properties of vericiguat. Specifically, vericiguat was found to reduce PAP to a modest extent and exhibited significant bronchodilator effects. These properties suggest that vericiguat does not worsen ventilation-perfusion mismatch, making it a promising candidate for further investigation in an animal model of PH related to COPD.

Endothelial dysfunction is a contributing factor to the pathogenesis of PH. The pro-inflammatory cytokines IL-6 and IL-1ß are considered to be key players in this process [2]. The absence of IL-6 expression exerts a protective effect on the development of PAH [4], while inhibition of the IL-1ß pathway delays the development of PH [38]. This study demonstrated that both riociguat and vericiguat significantly reduced the levels of IL-6 and IL-1ß. The findings are consistent with those of previous studies showing that riociguat or its analogue inhibits IL-6 release in the conjunctiva [39] and reduces IL-1β levels in a PH model [40]. The anti-inflammatory effects of vericiguat have not been described before and represent new properties of the drug. In conclusion, sGC stimulators may have the potential to delay the progression of PH through their anti-inflammatory properties.

Another significant contributor to the pathogenesis of PH is the chemokine KC/GRO (systematic name CXCL1), which is produced by resident macrophages or epithelial cells, activating neutrophils and initiating a cascade involving the release of pro-inflammatory cytokines [41,42]. Serum levels of CXCL1 correlate with mean PAP and C-reactive protein (CRP) in patients with PAH [43]. The impact of sGC stimulators on the chemokine levels remains poorly understood. In rats with bile duct ligation and portal hypertension, riociguat had no effect on CXCL1 [44]. Our results indicate that only riociguat reduces KC/GRO levels in isolated perfused rat lungs, whereas vericiguat had no effect. This phenomenon could be attributed to the observation that riociguat demonstrated greater efficacy in reducing PAP compared to vericiguat. Consequently, the reduction in shear stress may have resulted in a subsequent decrease in the release of KC/GRO. To our knowledge, this effect has not been described before.

Riociguat also showed a suppressive effect on VEGF-A. This is in contrast to studies showing that sGC stimulators increase VEGF [28,45], suggesting a context-dependent effect. In this study, the induction of vasoconstriction by endothelin-1 led to hypoxia, which, in turn, triggered the release of VEGF-A. After administration of riociguat and vericiguat, the drugs induced vasodilation, which improved blood flow and reduced hypoxia, leading to a decrease in VEGF-A levels. The mechanisms by which sGC stimulators regulate VEGF-A levels in diseased endothelium remain unclear. One hypothesis that may be postulated is that the administration of sGC stimulators results in the induction of the protein kinase G-dependent p44/p42 MAP kinase pathway [46], and the phosphorylation of hypoxia inducible factor alpha [47,48], thereby increasing VEGF-A and improving angiogenesis. The missing trigger for VEGF-A release (hypoxia) may have masked the underlying impact of riociguat on the PKG signaling pathway in the experiments. However, the mechanisms of suppression of cytokines and chemokines remained unaddressed. Given the three-hour duration of the experiment, only rapid immunological phenomena can be discerned. It is presumed that the release of pre-existing cytokines or chemokines can be quantified, but not gene regulation or expression changes. Further studies in animal models of PH caused by chronic hypoxia are necessary to verify this hypothesis.

This study has limitations. Isolated lung perfusion allows experiments to be performed on an intact organ without systemic side effects, with great control over ventilation and perfusion settings and drug concentration [49]. However, it is a very complex experimental setup with many influencing factors. An animal can be used for one experiment at a time, which includes the possibility that individual differences between animals may influence the results. The use of animals from the same tribe served to minimize the impact. The duration of the experiments was relatively short, with IPL lasting three hours and PCLS two hours. Pulmonary vasoconstriction was induced by the application of endothelin-1. This does not correspond to the full picture of pulmonary hypertension, which can develop over years in a complex process. As a result, the findings are only transferable to this disease to a limited extent.

Following the initial experimentation on healthy animals, future studies should confirm these effects in chronic PH models, in which pulmonary vascular remodeling occurs. Additionally, an investigation into vericiguat’s effects in an animal model of PH due to left heart disease would be of significant interest, as its benefits in the treatment of patients with reduced ejection fraction have been demonstrated. The improved characterization of vericiguat has provided valuable insights into its mechanisms of action and has identified new potential applications. The treatment of acute respiratory distress syndrome (ARDS) represents a potential clinical implication beyond that of PH. In models of ARDS, vericiguat could be tested as a new treatment option, as it reduces IL-6 and IL-1ß levels of endothelial inflammation and causes bronchodilation. The vasodilatory effect on the pulmonary arteries was small, suggesting that the use of vericiguat is unlikely to exacerbate ventilation-perfusion mismatch.

## 5. Conclusions

In summary, riociguat and vericiguat caused pulmonary artery dilation, bronchodilation, and acted as anti-inflammatories in two ex-vivo lung models of the rat. Clinical studies are needed to confirm their relevance in endothelial dysfunction. As demonstrated in other studies focusing on asthma or COPD, cGMP-mediated bronchodilation is a promising avenue for future therapeutic development. The anti-inflammatory properties appear to be an additional benefit in this context. In the future, vericiguat may emerge as a potential alternative to induce bronchodilation in the treatment of respiratory diseases.

## Figures and Tables

**Table 1:** Levels of significance for comparisons between riociguat, vericiguat, and control in pulmonary artery dilation in PCLS. GLMM analysis using a beta distribution was performed. Covariate analysis considered endothelin-1 and KCL effects. If a covariate affected the groups, an ANCOVA was used. Multiple comparisons were corrected with the SIM stepdown procedure. *p*-values < 0.05 were considered statistically significant * *p* < 0.05, ** *p* < 0.01, ns = not significant. Abbreviations are Rio = Riociguat, Ver = Vericiguat.

Treatment	*p*-Value (vs. Control)	*p*-Value (vs. Vericiguat)
1 µM Rio 10 µM Rio 32 µM Rio 1 µM Ver 10 µM Ver 32 µM Ver	* *p* = 0.041 * *p* = 0.049 ** *p* = 0.002 ns *p* = 0.79 ** *p* = 0.002 * *p* = 0.015	ns *p* = 0.15 ns *p* = 0.27 ns *p* = 0.49

**Table 2:** Significance levels for comparing riociguat, vericiguat, and control for bronchodilation; GLMM analysis was performed using a beta distribution. Endothelin-1 and KCL effects were considered. If a covariate affected the groups, an ANCOVA was used. Multiple comparisons were corrected with the SIM stepdown procedure. * *p* < 0.05, ** *p* < 0.01, *** *p* < 0.001, ns = not significant. Abbreviations are Rio = Riociguat, Ver = Vericiguat.

Treatment	*p*-Value (vs. Control)	*p*-Value (vs. Vericiguat)
1 µM Rio 10 µM Rio 32 µM Rio 1 µM Ver 10 µM Ver 32 µM Ver	** *p* = 0.001 *** *p* < 0.0001 *** *p* < 0.0001 ns *p* = 0.25 ** *p* = 0.003 *** *p* = 0.0005	* *p* = 0.045 ns *p* = 0.20 ns *p* = 0.36

**Table 3:** Significance levels for pulmonary artery dilation in isolated lung perfusion between riociguat, vericiguat, and control; GLMM analysis was performed on the absolute values, assuming a normal distribution. A covariate analysis considered the effect of endothelin-1. If the covariate affected the groups, an ANCOVA was performed. Multiple comparisons were corrected using the SIM step-down procedure. * *p* < 0.05, ** *p* < 0.01, ns = not significant. Abbreviations are Rio = Riociguat, Ver = Vericiguat.

Treatment	*p*-Value (vs. Control)	*p*-Value (vs. Vericiguat)
1 µM Rio 10 µM Rio 32 µM Rio 1 µM Ver 10 µM Ver 32 µM Ver	** *p* = 0.007 ** *p* = 0.001 ** *p* = 0.0095 ns *p* = 0.79 ns *p* = 0.054 * *p* = 0.017	** *p* = 0.007 * *p* = 0.034 ns *p* = 0.51

**Table 4:** Significant levels of airway resistance in IPL between riociguat, vericiguat, and control. GLMM analysis was performed on absolute values, assuming normal distribution. A covariate analysis took into account the effect of endothelin-1. If the covariate influenced the groups, an ANCOVA was performed. Multiple comparisons were corrected using the SIM step-down procedure. * *p* < 0.05, ns = not significant. Abbreviations are Rio = Riociguat, Ver = Vericiguat.

Treatment	*p*-Value (vs. Control)	*p*-Value (vs. Vericiguat)
1 µM Rio 10 µM Rio 32 µM Rio 1 µM Ver 10 µM Ver 32 µM Ver	ns *p* = 0.11 ns *p* = 0.17 ns *p* = 0.33 * *p* = 0.049 * *p* = 0.039 * *p* = 0.018	ns *p* = 0.29 ns *p* = 0.15 * *p* = 0.029

**Table 5:** Significance levels of differences in cytokine suppression after treatment with riociguat or vericiguat compared to control. A GLMM analysis was performed assuming a normal distribution with degrees of freedom adjusted for heteroscedasticity. A covariate analysis was performed to account for the effect of endothelin-1 on the results. If the covariate had an effect, an ANCOVA was performed, and multiple comparisons were corrected using the SIM stepdown procedure. * *p* < 0.05, ** *p* < 0.01, ns = not significant.

Cytokine	Treatment	*p*-Value (vs. Control)	*p*-Value (vs. Vericiguat)
VEGF-A	riociguat	* *p* = 0.039	* *p* = 0.048
	vericiguat	ns *p* = 0.52	
KC-GRO	riociguat	** *p* = 0.0028	** *p* = 0.0066
	vericiguat	ns *p* = 0.51	
IL-6	riociguat	* *p* = 0.03	ns *p* = 0.71
	vericiguat	* *p* = 0.03	
IL-1ß	riociguat	* *p* = 0.037	ns *p* = 0.49
	vericiguat	* *p* = 0.026	

## Data Availability

The original contributions presented in this study are included in the article/Supplementary Material. Further inquiries can be directed to the corresponding author.

## Supplementary Materials

The following supporting information can be downloaded at: https://www.mdpi.com/article/10.3390/biomedicines13040856/s1, Table S1: Significance levels of differences in suppression of the cytokines IL-4, IL-5, IL-10, IL-13, TNFα, MCP-1, or IFNγ after treatment with riociguat or vericiguat compared to control.

## Abbreviations

The following abbreviations are used in this manuscript:

ARDS	Acute respiratory distress syndrome
cGMP	Cyclic guanosine monophosphate
COPD	Chronic obstructive pulmonary disease
CRP	C-reactive protein
CTEPH	Chronic thromboembolic pulmonary hypertension
CXCL1	Chemokine (C-X-C motif) ligand 1
DMSO	Dimethyl sulfoxide
eNOS	Endothelial nitric oxide synthase
IAA	Initial airway area
IFNγ	Interferon γ
IL-10	Interleukin-10
IL-13	Interleukin-13
IL-4	Interleukin-4
IL-5	Interleukin-5
IL-1ß	Interleukin-1ß
IL-8	Interleukin-8
IL-6	Interleukin-6
IPL	Isolated lung perfusion
IVA	Initial vessel area
PVR	Pulmonal vascular resistance
KC-GRO	Keratinocyte chemoattractant/human growth-regulated oncogene
MCP-1	Monocyte chemoattractant protein-1
NO	Nitric oxide
PAH	Pulmonary arterial hypertension
PAP	Pulmonary artery hypertension
PCLS	Precision Cut Lung Slices
PH	Pulmonary Hypertension
PKG	Protein Kinase G
Rio	Riociguat
SEM	Standard error of the mean
sGC	Soluble guanylate cyclase
TNFα	Tumor necrosis factor α
VEGF-A	Vascular endothelial growth factor A
Ver	Vericiguat
