# Supplementary material for: Different Effects of Riociguat and Vericiguat on Pulmonary Vessels and Airways
Source: Biomedicines. 2025 Apr 2;13(4):856. doi: 10.3390/biomedicines13040856 (PMC12024824; doi:10.3390/biomedicines13040856)
Supplement: Supplementary file 1 [file biomedicines-13-00856-s001.zip › biomedicines-3512800-supplementary.pdf]

Supplementary

Different effects of Riociguat and Vericiguat on pulmonary vessels and airways

Katharina Nubbemeyer[1], Julia Krabbe[2], Svenja Böll[3,4], Anna Michely[3,4], Sebastian Kalverkamp[1], Jan Spillner[1], Christian Martin[4*]

Table S1. Significance levels of differences in suppression of the cytokines IL-4, IL-5, IL-10, IL13, TNFα, MCP-1 or IFNγ after treatment with riociguat or vericiguat compared to control; all p>0,05 with no statistical significance.

Cytokine	Treatment	p-value (vs. control)	p-value (vs. vericiguat)
MCP-1	riociguat	0.63	0.90
	vericiguat	0.63	
TNFα	riociguat	0.48	0.80
	vericiguat	0.48	
IL-4	riociguat	0.07	0.90
	vericiguat	0.07	
IL-5	riociguat	0.40	0.59
	vericiguat	0.43	
IL-10	riociguat	0.16	0.66
	vericiguat	0.16	
IL-13	riociguat	0.59	0.78
	vericiguat	0.59	
IFNγ	riociguat	0.90	0.90
	vericiguat	0.90	
